# The double-edged sword effect of estrogen in kidney disease and its precision modulation

**DOI:** 10.3389/fendo.2026.1819314

**Published:** 2026-06-18

**Authors:** Jinlan Zhang, Jiayi Lyu, Miao Deng, Jinfen Han, Qin Wang, Jia Song, Rubin Zheng, Zhixun Bai

**Affiliations:** 1 People’s Hospital of Qianxinan Prefecture, Xingyi, Guizhou, China; 2 Zunyi Medical University, Zunyi, Guizhou, China

**Keywords:** double-edged sword effect, estrogen, kidney disease, precision modulation, sexual dimorphism

## Abstract

Estrogen, a critical sex hormone, exerts a complex dual role in renal physiology and pathology. While it confers protective effects through anti-inflammatory, anti-fibrotic, and hemodynamic-related signaling pathways—slowing chronic kidney disease (CKD) progression and improving renal function—it may also promote disease under specific pathological contexts. Sexual dimorphism, mediated primarily by estrogen receptor (ER) signaling networks, plays a pivotal role in this duality. This review focuses on the molecular mechanisms of estrogen in kidney disease, the influence of sexual dimorphism, and the double-edged nature of its effects, with an emphasis on precision modulation strategies. By integrating recent basic and clinical findings, we analyze the diverse roles of estrogen across kidney disease models and evaluate its therapeutic potential. Furthermore, we discuss the prospects of applying sex-based precision medicine to kidney disease management, aiming to provide theoretical foundations and guidance for future research and clinical practice.

## 1 Introduction

Chronic kidney disease (CKD) have become a major threat to global health. According to the Global Burden of Disease Study (GBD) 2023, the age-standardized prevalence of CKD among adults aged ≥20 years was 14.2% in 2023. The global CKD patient population is estimated to have reached 788 million, a significant increase from 378 million in 1990. Furthermore, CKD was the ninth leading cause of death globally in 2023, accounting for 1.48 million deaths, and the twelfth leading cause of disability-adjusted life years (DALYs), with an age-standardized DALY rate of 769.2 per 100,000 people (1). Based on Autoregressive Integrated Moving Average (ARIMA) model predictions, by 2031, the number of chronic kidney failure cases is projected to rise to 22.21 million, and the number of deaths to rise to 1.81 million (2).

One of the most striking features of CKD epidemiology is sexual dimorphism: premenopausal women generally exhibit slower renal function decline than age−matched men, suggesting that female sex hormones, particularly estrogen, may play a protective role. In addition to its role in reproductive function, estrogen is also crucial for renal structure. However, the complex dual nature of its actions has not yet been fully elucidated.

Recent basic and clinical research has revealed multifaceted mechanisms of estrogen in kidney diseases, including anti−inflammation, anti−oxidation (3), anti−fibrosis (4), and regulation of tubular and vascular function (5). Estrogen and its receptors are involved in diverse renal physiological processes: maintenance of mitochondrial homeostasis (6), regulation of the endothelin−1 system (7), participation in renal repair and regeneration (8), and modulation of proximal tubular phosphate homeostasis (9). Polymorphisms in the estrogen receptor α (ERα) gene are associated with susceptibility to and prognosis of various kidney diseases (6, 10). Although targeting estrogen/receptor signaling pathways may offer renoprotective benefits, the precise mechanisms in different disease contexts remain largely unclear, necessitating further investigation for the development of targeted therapies.

Fluctuations in sex hormone levels, particularly the decline in estrogen in perimenopausal women, are closely associated with an increased incidence of chronic kidney disease. A 15-year prospective cohort study involving over 3,000 women found that those with a shorter duration of endogenous estrogen exposure had a significantly higher risk of developing CKD later in life, suggesting a negative correlation between estrogen exposure time and CKD onset (11). Moreover, renal function markers such as blood urea nitrogen and creatinine change dynamically in perimenopausal and postmenopausal women in a manner linked to estrogen levels (12), further supporting a renoprotective role of estrogen.

Beyond anti−inflammatory and antioxidant effects, estrogen regulates renal ion channels and aquaporins. For instance, estrogen modulates epithelial sodium channel (ENaC) activity via the G protein−coupled estrogen receptor (GPER), promoting female−specific natriuresis and thereby maintaining extracellular fluid volume and blood pressure homeostasis (13, 14). This has important implications for preventing hypertension and cardiovascular disease, particularly in women.

Conversely, under certain pathological conditions, estrogen may promote kidney disease progression. Elevated estrogen levels during pregnancy can increase liver volume and hepatic cyst size, suggesting that estrogen contributes to cyst expansion by stimulating cell proliferation (15). In autoimmune−prone female mice, estrogen exposure enhances immune responses and exacerbates lupus nephropathy (16) (see Section 4.3 for details).

Thus, estrogen exerts a double−edged sword effect in kidney diseases. The molecular mechanisms that determine whether estrogen acts as a friend or foe in different renal conditions remain poorly defined. A systematic elucidation of the signaling pathways, receptor crosstalk, and context−dependent factors is urgently needed to guide the development of precision modulation strategies, thereby providing theoretical support and identifying novel therapeutic targets for clinical management.

## 2 Fundamental mechanisms of estrogen in renal physiological function

### 2.1 Characteristics of estrogen synthesis and metabolism in the kidney

The synthesis of estrogen primarily depends on the classic steroid hormone biosynthesis pathway, with core steps involving the conversion of cholesterol to pregnenolone, followed by a series of enzymatic reactions that yield major active estrogen forms such as estradiol (E2) (17). Although preliminary evidence suggests the potential for estrogen biosynthesis within the distal nephron of the kidney (18), this concept has not been fully elucidated in broader clinical or high-quality experimental studies.

The kidney plays an important role in estrogen metabolism. It mediates hydroxylation and sulfonation reactions of estrogen, generating various estrogen metabolites such as estrone, estriol, and their sulfated forms, which exert distinct biological effects locally within the kidney. The sulfonated products of estrogen serve a buffering role in regulating local hormone activity and protecting renal tissue from excessive hormonal stimulation (19). By binding to estrogen receptors (ERs), these metabolites modulate renal cell proliferation, apoptosis, and inflammatory responses, thereby influencing the progression and repair processes of kidney diseases (20).

Recent metabolomic studies have revealed significant alterations in the expression of estrogen and its metabolic enzymes in patients with CKD. Particularly during pathological processes such as vascular calcification, enzymes involved in estrogen synthesis—including steroid sulfatase and estrogen sulfotransferase—are downregulated, leading to impaired local estrogen metabolism in the kidney and consequently exacerbating disease progression (19).

### 2.2 Expression and function of estrogen receptors (ERα, ERβ, GPER) in the kidney

ERs primarily include the classical nuclear receptors ERα and ERβ (estrogen receptor β, ERβ), as well as the non-classical transmembrane receptor GPER. The distribution characteristics of these three receptors in the kidney and their respective signal transduction pathways are crucial for regulating renal cell function.

In terms of receptor distribution, ERα and GPER are the predominant estrogen receptor types in the kidney and other cardiovascular tissues, whereas ERβ expression is more limited and primarily detectable in the kidney. Animal model studies indicate that ERα and GPER exhibit high mRNA expression levels in tissues such as the kidney, heart, and aorta. Notably, female animals show significantly higher ERα and GPER expression in these organs than males, revealing marked sex differences. In contrast, ERβ expression is relatively low and highly tissue-specific (21). Expression patterns also vary across different renal cell types; for instance, ERα and GPER are abundant in renal tubular cells, where they participate in regulating water-salt metabolism and inflammatory responses (22, 23).

In signal transduction, ERα and ERβ function as nuclear receptors, primarily mediating transcriptional regulation within the nucleus to control renal cell functions such as metabolism, proliferation, and apoptosis. ERα modulates insulin-like growth factor 1 receptor expression in proximal tubule (PT) cells, thereby influencing cell cycle progression and susceptibility to ischemia-reperfusion injury (24). ERβ participates in regulating fatty acid oxidation (FAO) and extracellular matrix (ECM) synthesis, with its deficiency or abnormal expression closely associated with the progression of renal fibrosis (25).

GPER, a seven-transmembrane receptor located on the cell membrane, mediates rapid non-genomic estrogen signaling, regulating calcium ion flux, cell proliferation, and antioxidant responses. In models of Acute kidney injury (AKI), GPER expression correlates with estrogen’s protective effects on the kidneys. GPER agonists reduce apoptosis and oxidative stress while promoting renal function recovery (26, 27). Notably, GPER expression and function may vary with age and sex. Female kidneys exhibit higher GPER expression and active function, contributing to the regulation of vasodilation and anti-inflammatory responses (21).

ER-mediated signaling pathways interact with other signaling networks. ERβ synergistically regulates fatty acid metabolism gene expression with peroxisome proliferator-activated receptor α (PPARα), influencing renal energy metabolism and fibrotic processes (25). GPER also participates in protecting against AKI by regulating endoplasmic reticulum stress and the TLR4/NF-κB/NLRP3 inflammatory pathway (22, 26). These intricate signaling networks enable estrogen receptors to exert multidimensional regulatory effects in both physiological and pathological conditions within the kidney.

A deeper understanding of the expression dynamics of these three receptors and their mediated signaling mechanisms is crucial. This intricate network of ERα, ERβ, and GPER signaling directly impacts glomerular and tubular function, forming the basis for the mechanisms discussed in the following section.

### 2.3 Mechanisms of estrogen regulation of glomerular filtration rate and tubular function

Epidemiological and clinical observations consistently indicate a sexual dimorphism in CKD progression, with females often exhibiting a more favorable prognosis than males. This disparity strongly implicates sex hormones, particularly estrogen, in conferring renal protection (14, 28). Estrogen primarily mediates its effects via estrogen receptors (especially ERα), which are expressed in both glomerular and tubular compartments, thereby modulating renal hemodynamics and metabolic processes (29).

A cornerstone of estrogen’s renal protective action is its modulation of glomerular hemodynamics, largely achieved through the endothelial nitric oxide synthase (eNOS)-nitric oxide (NO) pathway (30). In renal vascular endothelial and mesangial cells, estrogen binding to ERα triggers both genomic and rapid non−genomic signaling. Genomically, estrogen upregulates eNOS expression (30); through rapid non−genomic pathways, it enhances eNOS phosphorylation (activation) (31), significantly boosting NO production. Notably, membrane−associated ERα itself can act as a direct sensor of fluid shear stress. It can be activated in a ligand−independent manner to initiate the eNOS−NO signaling pathway (29). As a potent vasodilator, NO activates soluble guanylate cyclase in vascular smooth muscle, elevating cyclic guanosine monophosphate (cGMP) levels. The resultant relaxation of afferent and efferent arterioles reduces renal vascular resistance, increases renal blood flow, and helps stabilize the glomerular filtration rate (GFR) (14).

Clinical and experimental evidence supports this mechanism. For instance, in patients with immunoglobulin A nephropathy (IgAN), renal ERα expression positively correlates with estimated GFR, underscoring the importance of estrogen signaling in preserving filtration function (32). Consistently, interventional studies indicate that estrogen therapy increases renal blood flow and GFR, whereas testosterone may exert opposing effects (33).

In addition to regulating glomerular hemodynamics via the eNOS−NO pathway, estrogen directly targets the renal tubules to modulate sodium and water reabsorption, thereby contributing to hemodynamic homeostasis. Several key transporters have been identified as molecular targets of estrogen. With respect to the ENaC, estrogen downregulates its activity through Derlin−1 and AMPK (34). Notably, this effect is mediated in a female−specific manner by GPER1, as recently demonstrated using a GPER1 agonist (13). For the Na^+^−K^+^−2Cl^-^ cotransporter type 2 (NKCC2) and the sodium−chloride cotransporter (NCC), estrogen reduces their renal expression (35); however, the specific estrogen receptor subtypes responsible for these actions remain to be identified. Regarding aquaporin−2 (AQP2), estrogen suppresses its gene transcription via estrogen receptor α (ERα), leading to decreased AQP2 protein synthesis and reduced water reabsorption (23, 36). In addition, estrogen may influence these transporters through indirect mechanisms, such as transcriptional regulation via estrogen response elements or alterations in plasma potassium concentration (37, 38). Collectively, these findings indicate that estrogen coordinately regulates multiple tubular transporters and channels, thereby alleviating sodium load, preserving tubular integrity, and maintaining fluid and blood pressure homeostasis (39).

Thus, estrogen safeguards renal function through a dual mechanism: sustaining glomerular perfusion via the eNOS−NO pathway and fine−tuning tubular sodium reabsorption (14). This integrated regulation of filtration and reabsorption highlights estrogen’s pivotal role in renal physiology. Targeting these pathways with selective estrogen receptor modulators (SERMs) may offer a promising therapeutic avenue for mitigating kidney disease progression, particularly in estrogen−deficient states.

## 3 The dual role of estrogen in chronic kidney disease

### 3.1 Renoprotective effects of estrogen: anti-inflammatory and anti-fibrotic mechanisms

The protective role of estrogen in kidney disease primarily manifests through anti-inflammatory and anti-fibrotic effects. Regarding anti-inflammation, estrogen effectively mitigates kidney injury by suppressing the expression of pro-inflammatory cytokines and oxidative stress responses. Relevant studies indicate that 17β-E2 reduces levels of multiple pro-inflammatory factors such as tumor necrosis factor-α (TNF-α), interleukin-1β (IL-1β), and interleukin-6 (IL-6), thereby attenuating renal inflammatory responses and protecting kidney function. In the lipopolysaccharide (LPS)−induced AKI model, ERα and ERβ jointly mediate the anti−inflammatory protective effects of estrogen—both are indispensable and act synergistically (40). *In vitro* macrophage studies suggest that GPER1 activation suppresses LPS−induced inflammatory responses: the GPER agonist G−1 reduces LPS−induced IL−6 expression by inhibiting NF−κB promoter activity (41); GPER1 activation also reduces macrophage polarization toward the pro−inflammatory M1 phenotype and impairs inflammatory signaling upon LPS/IFN−γ stimulation (42). These *in vitro* findings suggest that GPER1 may contribute an auxiliary anti−inflammatory role via rapid non−genomic mechanisms, although direct *in vivo* validation in LPS−AKI models is still needed. Estrogen also modulates immune cell function by balancing the T helper 17/regulatory T cell (Th17/Treg) ratio, further suppressing immune-mediated renal inflammation (43). Regarding oxidative stress, estrogen enhances antioxidant capacity by activating molecules such as sirtuin 1, thereby reducing reactive oxygen species (ROS) production and mitigating oxidative damage and apoptosis (44, 45).

In terms of anti-fibrosis, estrogen inhibits the progression of renal fibrosis by negatively regulating fibrosis-related signaling pathways. One core mechanism of renal fibrosis involves activation of the transforming growth factor-β (TGF-β)/Smad signaling pathway, which induces expression of fibrosis-related genes and promotes excessive ECM deposition. Research indicates that activation of ERβ competitively inhibits Smad3 binding to target gene promoters, reducing transcription of fibrotic genes without affecting Smad3 phosphorylation status, thereby alleviating renal fibrosis (46). *In vitro* studies have shown that activation of GPER1 reduces the polarization of M0 macrophages toward either the M1 or M2 phenotype. Activation of GPER1 in M1 macrophages was found to impair inflammatory signaling, thereby protecting tubular epithelial cells (TECs) from immune activation and injury. Furthermore, activation of GPER1 in M2 macrophages inhibits the transition of resident fibroblasts into myofibroblasts, suppressing inflammatory and fibrotic responses (42). Phytoestrogenic compounds such as kaempferol and quercetin exhibit similar antifibrotic effects, partially achieved by modulating TGF-β signaling and oxidative stress-related pathways (47, 48).

Both clinical and experimental studies indicate that estrogen and its receptors exert crucial protective effects in female kidney diseases, particularly in acute and chronic kidney injury and diabetic nephropathy. Estrogen slows renal functional decline, reduces proteinuria and fibrosis markers, and maintains stable renal structure and function through multiple mechanisms (49, 50). SERMs such as raloxifene and tamoxifen also demonstrate potent protective effects in renal ischemia−reperfusion injury models, supporting therapeutic strategies that target estrogen signaling for renal protection (51, 52).

**Figure 1:**
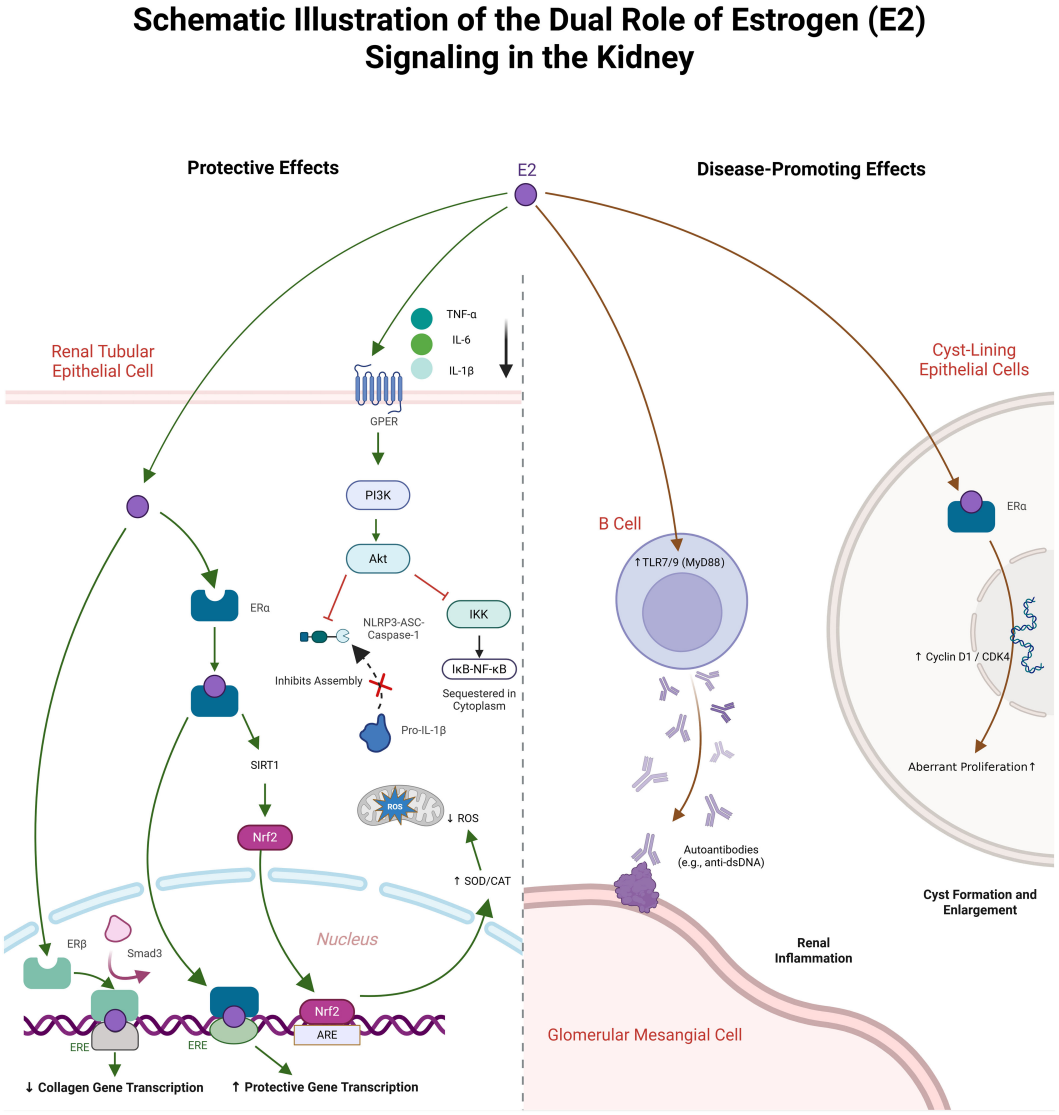
Schematic illustration of the dual role of estrogen (E2) signaling in the kidney. This diagram summarizes the context-dependent mechanisms by which E2 exerts opposing effects on renal physiology. Left panel (Protective Effects): E2 confers renoprotection through distinct receptor-mediated pathways. (1) Anti-inflammatory pathway: E2 binding to GPER activates the PI3K/Akt/IKK axis, leading to cytoplasmic sequestration of IκB-NF-κB and inhibition of NLRP3 inflammasome assembly, thereby reducing pro-inflammatory cytokines (e.g., TNF-α, IL-6, IL-1β). (2) Anti-fibrotic & Antioxidant pathways: E2 binding to nuclear receptors ERα/β enhances SIRT1/Nrf2 signaling, which suppresses Smad3-driven collagen gene transcription and upregulates antioxidant enzymes (SOD, CAT) to scavenge ROS. Right panel (Disease-Promoting Effects): In specific pathological contexts, E2 promotes disease progression through distinct receptors: (1) In polycystic kidney disease, E2-ERα signaling in cyst-lining epithelial cells drives aberrant proliferation via upregulation of Cyclin D1/CDK4, leading to cyst formation and enlargement. (2) In autoimmune nephropathy, E2 via GPER potentiates B cell TLR7/9-MyD88 signaling, leading to autoantibody (e.g., anti-dsDNA) production and subsequent immune complex glomerulonephritis. Created in https://BioRender.com.

Together, these anti−inflammatory and anti−fibrotic mechanisms define a core protective axis of estrogen signaling in the kidney (**Figure 1**, left panel), providing a theoretical basis for precisely modulating this pathway to achieve renoprotection (40, 46, 53).

### 3.2 Potential mechanisms of estrogen in promoting pathological progression

Estrogen exerts complex bidirectional effects in kidney disease. While it can confer protection, it may also accelerate disease progression under specific pathological conditions. The pro−pathological mechanisms primarily involve induction of aberrant cell proliferation, triggering of oxidative stress and endothelial dysfunction, and immune dysregulation mediated by estrogen receptor signaling.

Aberrant proliferation and cyst formation. In patients with autosomal dominant polycystic kidney disease (ADPKD), elevated maternal estrogen levels during pregnancy correlate with increased cyst volume in the liver and kidneys, suggesting that estrogen exacerbates cyst progression by promoting proliferation of cyst−lining epithelial cells (15). Mechanistically, estrogen binding to ERα upregulates cyclins (Cyclin D1, CDK4), driving excessive proliferation of these cells (54).

Oxidative stress and endothelial/immune dysregulation. Estrogen and its analogues can accumulate in renal cells, triggering oxidative stress cascades that include mitochondrial dysfunction and excessive reactive oxygen species production. These events lead to podocyte apoptosis, thickening of the glomerular basement membrane, myofibroblast activation, and extracellular matrix deposition, thereby directly aggravating glomerular and interstitial injury and accelerating renal fibrosis (55). In addition, aberrant estrogen signaling may promote chronic kidney disease progression by influencing renal immune cell infiltration and local release of inflammatory cytokines (14).

Estrogen receptor−mediated autoimmune kidney injury. In autoimmune kidney diseases such as systemic lupus erythematosus (SLE), estrogen clearly promotes disease progression, largely through estrogen receptor subtypes, especially GPER and ERα. On the one hand, estrogen enhances Toll−like receptor 7/9 (TLR7/9) signaling in B cells via a MyD88−dependent pathway, promoting B cell differentiation into plasma cells and the production of large amounts of autoantibodies (e.g., anti−dsDNA). These autoantibodies form immune complexes that deposit in the glomeruli, triggering immune complex−mediated glomerulonephritis (16). On the other hand, estrogen upregulates renal platelet−activating factor acetylhydrolase (PAF−AH) activity, affecting the metabolism of inflammatory mediators and further exacerbating tissue injury (56). Together, these receptor−specific mechanisms constitute the core molecular basis by which estrogen promotes the progression of autoimmune kidney disease.

Collectively, the above mechanisms reveal the disease−promoting effects of estrogen in kidney diseases, highlighting the harmful side of its double−edged sword (**Figure 1**, right panel).

## 4 Estrogen and specific kidney diseases

### 4.1 The role of estrogen in diabetic kidney disease

Diabetic kidney disease (DKD) is one of the most common and severe complications of diabetes, characterized by pathological changes in the glomeruli and renal tubulointerstitium, and may ultimately progress to end−stage renal disease (ESRD). During the progression of DKD, estrogen and its receptors exert complex regulatory effects, primarily protecting renal function through mechanisms such as improving glucose metabolism, alleviating oxidative stress, and maintaining mitochondrial function. However, the renoprotective effects of estrogen in DKD are modulated by diabetes type, sex, hormonal balance, and coexisting clinical conditions, exhibiting a complexity that distinguishes DKD from other chronic kidney diseases.

Unlike other CKDs, where male sex consistently portends a faster decline in renal function (57), whether men are at greater risk for progressive kidney function loss in DKD has not been definitively established. Evidence concerning sex differences in DKD epidemiology remains fragmented and critically depends on diabetes type as well as the stage of kidney involvement. In type 1 diabetes, some studies report accelerated nephropathy progression and higher end−stage renal disease incidence in men (58), whereas others indicate that women are more prone to developing microalbuminuria (59). This apparent paradox suggests that estrogen’s proposed renoprotection may operate primarily by retarding the transition from incipient to overt kidney damage, rather than by preventing the initial appearance of albuminuria (8). In type 2 diabetes, the relationship becomes even less straightforward. The greater diversity of background factors, including hypertension, cardiovascular comorbidity, and prior medication regimens, frequently confounds the analysis, and their complex interactions with sex hormones can either mask or supersede any inherent sex−based effect (60, 61). Consequently, the association between sex and DKD outcomes in type 2 diabetes proves far less consistent than in other kidney diseases. These epidemiological complexities collectively caution against directly extrapolating the conventional paradigm of universal female renoprotection to DKD. Instead, any interpretation of estrogen’s role in the diabetic kidney must incorporate systematic consideration of diabetes type, metabolic context, and coexisting clinical conditions (8, 62, 63).

Estrogen exerts renoprotective effects by regulating glucose metabolism and alleviating oxidative stress. Estrogen improves insulin sensitivity, modulates blood glucose levels, and mitigates renal damage caused by hyperglycemia. ERα and ERβ expressed in renal cells mediate this protective effect (8) and inhibit the progression of glomerulosclerosis and renal interstitial fibrosis by regulating downstream signaling pathways. Moreover, plant polyphenols such as resveratrol exhibit estrogen−like activity and may provide adjunctive therapeutic benefits through anti−inflammatory and antioxidant mechanisms (64). At the cellular and molecular level, ERα36, an estrogen receptor variant, exerts protective effects under hyperglycemic conditions by activating the phosphatidylinositol 3-kinase/protein kinase B (PI3K/AKT) signaling pathway, thereby inhibiting apoptosis and senescence in renal tubular cells and mitigating cellular damage and renal dysfunction (65). Estrogen−related receptor α (ERRα) plays a crucial role in maintaining mitochondrial function in renal tubular cells; its downregulation is closely associated with mitochondrial damage in DKD. Modulating ERRα expression enhances mitochondrial biogenesis and FAO, improves metabolic abnormalities, and alleviates renal injury (66, 67). Sodium-glucose cotransporter 2 (SGLT2) inhibitors such as dapagliflozin exert renoprotective effects by upregulating ERRα and related enzymes (e.g., ACOX1), suggesting that the ERRα pathway is a critical therapeutic target for DKD (68).

Beyond the regulatory effects of estrogen itself, a broader and more integrated paradigm has emerged specifically for DKD: both male and female patients with DKD exhibit a systemic imbalance between blood testosterone and estrogen, and correcting this imbalance suppresses nephropathy progression. Clinical studies have demonstrated altered testosterone−to−estradiol ratios in men with type 1 diabetes and nephropathy (69); fertile women with type 1 diabetes also display distinctive endocrine profiles that correlate with renal outcomes (70). Animal studies directly demonstrated that an imbalance in sex hormone levels exacerbates diabetic renal disease and that restoring hormonal equilibrium—instead of manipulating a single sex hormone—effectively attenuates renal injury (71, 72). Mechanistically, an unfavorable testosterone/estrogen balance promotes renal inflammation, fibrosis, and podocyte injury through intersecting pathways involving the renin−angiotensin system, transforming growth factor−β, and oxidative stress (73, 74). From a therapeutic perspective, these findings shift the focus from estrogen replacement or estrogen receptor modulation alone toward restoring a protective hormone balance, a strategy with direct implications for both female and male DKD patients (8). Thus, in DKD, the net renal outcome is determined not by estrogen in isolation but by the integrated balance of estrogen receptor and androgen receptor signaling.

### 4.2 Regulation of estrogen in hypertension-related nephropathy

The regulatory role of estrogen in hypertension-related Nephropathy primarily manifests through its effects on the RAAS and the resulting renal protective effects. The RAAS is a key system regulating blood pressure stability and fluid balance, and its abnormal activation is one of the core mechanisms underlying hypertension and renal injury. Research indicates that estrogen modulates RAAS activity through multiple pathways, thereby exerting antihypertensive and renal protective effects. Specifically, estrogen can inhibit the activity of the classic angiotensin II -angiotensin II type 1 receptor axis activity, reducing vasoconstriction and sodium/water retention. Concurrently, it promotes the expression of protective angiotensin-(1-7) and its receptor Mas, enhancing vasodilation and anti-inflammatory responses (55). Furthermore, estrogen modulates the expression of renal local RAS components, reducing glomerular hypertension and fibrosis while delaying renal function decline (75). Beyond classical ERα and ERβ, this regulation also involves GPER, whose activation promotes antioxidant and anti-inflammatory signaling pathways, effectively mitigating hypertensive renal damage (76).

Sex differences are particularly pronounced in hypertension-induced renal injury. Both clinical and animal models demonstrate that females with normal physiological estrogen levels exhibit lower blood pressure and reduced susceptibility to renal damage, closely linked to estrogen’s regulation of the RAAS. Estrogen signaling in female kidneys supports self-renewal and differentiation of renal progenitor cells, enhances glomerular filtration function, and thereby increases renal resistance to injury (77). In contrast, males and postmenopausal females exhibit elevated blood pressure and increased renal vulnerability to injury due to decreased estrogen levels and heightened RAAS activity, manifesting as glomerulosclerosis, increased proteinuria, and renal function decline (78, 79). Research further reveals that under estrogen-deficient conditions, impaired renal mitochondrial function and heightened inflammatory responses accelerate the progression of hypertensive nephropathy (79, 80). This suggests that considering patient sex and hormonal status to implement precise estrogen regulation may improve outcomes in treating hypertension-related kidney disease.

### 4.3 Estrogen and autoimmune kidney disease

#### 4.3.1 Disease−promoting mechanisms

The incidence of autoimmune kidney disease is significantly higher in females than in males, which is closely related to the immunomodulatory effects of estrogen. Physiologic levels of estrogen promote B−cell activation, autoantibody production, and activation of innate immune pathways. Studies have shown that the synthetic estrogen 17α−ethinyl estradiol enhances B−cell activity and promotes the production of anti−dsDNA antibodies by modulating the response to Toll−like receptor 7/9 (TLR7/9) agonists, leading to glomerular immune complex deposition and kidney injury (16). Furthermore, estrogen upregulates the expression of TLR7 and TLR8, facilitating the release of pro−inflammatory microRNAs (e.g., miR−21, miR−29a, and miR−29b) via exosomes, which further activates inflammatory pathways, triggers immune cell dysfunction, and causes tissue damage (81).

#### 4.3.2 Protective mechanisms

Conversely, estrogen can also exhibit protective effects in autoimmune kidney disease. In adult mouse models of SLE, estrogen removal does not confer protection but rather exacerbates SLE−associated hypertension and kidney injury (82–85). For example, ovariectomy performed on 4−week−old MRL/+ mice led to elevated serum anti−dsDNA antibody levels and accelerated the onset of autoimmune lesions (86). Similarly, in MRL/LPR mice, ovariectomy resulted in earlier development of severe autoimmune arthritis, and subsequent estrogen administration reversed these autoimmune lesions (87). Mechanistically, estradiol has been shown to upregulate eNOS while suppressing inducible nitric oxide synthase (iNOS) in immune cells, thereby preventing the detrimental effects of excessive nitric oxide produced by activated macrophages (88). This mechanism is particularly important in inflammatory glomerulonephritis, where activated macrophages play a key role (88). In addition, estrogen has been demonstrated to ameliorate T−cell−mediated renal vasculitis in certain autoimmune models (89).

Thus, estrogen exerts a “double−edged sword” effect in autoimmune kidney disease: it promotes the disease through B−cell activation and TLR signaling pathways, while providing protection through the eNOS/iNOS/NO pathway and maintenance of immune homeostasis. The net effect of estrogen is highly dependent on the specific immune context, disease stage, and hormonal balance. Understanding this dual role is critical for the development of safe and effective targeted therapeutic strategies.

## 5 Estrogen-related signaling pathways and their precision regulation strategies

### 5.1 Complexity and cross-regulation of the estrogen receptor signaling network

The estrogen receptor signaling network exhibits remarkable complexity, primarily manifested in the interactions among different types of estrogen receptors and their cross-regulation with other nuclear receptors. As summarized in **Table 1**, estrogen exhibits dual effects across various kidney diseases, exerting protective roles in certain contexts while promoting disease progression in others, which is a direct reflection of its intricate signaling network. The estrogen receptor family primarily includes the classic nuclear receptors ERα and ERβ, as well as the membrane-bound GPER. These three receptors are characterized by differences in tissue distribution and also mediate signaling pathways with distinct regulatory mechanisms and biological functions.

**Table 1:** Summary of the context-dependent dual roles and underlying mechanisms of estrogen (E2) in the kidney.

Aspect	Protective effects	Disease-promoting effects
Primary Context	Homeostatic conditions or the early phase of injury	Specific pathological contexts: Polycystic Kidney Disease (PKD), Autoimmune Nephropathy, and states of Metabolic Stress (e.g., Hyperglycemia)
Main Receptor(s)	Nuclear Estrogen Receptors (ERα, ERβ)	PKD: ERα Autoimmune Nephropathy: GPER Metabolic Stress: GPER
Key Signaling Pathways/Mechanisms	1. Anti-inflammatory: Inhibits NF-κB activation via the PI3K/Akt/IKK axis. Suppresses NLRP3 inflammasome assembly. 2. Anti-fibrotic & Antioxidant: Activates the SIRT1/Nrf2 pathway. Attenuates Smad3-mediated signaling. Upregulates antioxidant enzymes (SOD, CAT).	PKD: The E2-ERα complex promotes aberrant epithelial proliferation by upregulating cell cycle regulators (Cyclin D1, CDK4). Autoimmune Nephropathy:E2, via GPER, enhances TLR7/9-MyD88 signaling in B cells, leading to autoantibody (e.g., anti-dsDNA) production. Metabolic Stress: E2-GPER signaling synergizes with high glucose to exacerbate oxidative stress (via upregulation of NOX4) and ER stress (via CHOP induction).
Core Effector Molecules	↑: HSPs, SIRT1, Nrf2, SOD, CAT ↓: Nuclear NF-κB, NLRP3, IL-1β, IL-6, TNF-α, Smad3	PKD: ↑Cyclin D1, CDK4 Autoimmunity: ↑TLR7/9, autoantibodies Metabolic Stress: ↑NOX4, CHOP
Final Renal Outcome	Attenuation of inflammation, reduction of oxidative damage, and suppression of fibrosis.	PKD: Cyst formation and enlargement. Autoimmunity: Immune complex deposition and glomerulonephritis. Metabolic Stress: Tubular cell apoptosis and progression of renal fibrosis.

As classic nuclear transcription factors, ERα and ERβ regulate various physiological processes such as cell proliferation, differentiation, and metabolism by controlling gene transcription. ERα is typically associated with cell proliferation and tumor promotion, whereas ERβ predominantly exhibits cell growth-inhibitory functions (90). The ratio and balance between these two receptors significantly influence cellular physiological states. In hormone-dependent diseases such as breast cancer, alterations in the expression ratio of ERα and ERβ are closely linked to disease progression and therapeutic response (91, 92).

GPER, as a membrane-bound seven-transmembrane G protein-coupled receptor, mediates non-genomic rapid signal transduction, regulating intracellular second messengers and kinase cascades. GPER is expressed in multiple tissues and plays crucial regulatory roles in the nervous system, cardiovascular system, and tumor microenvironment (93, 94). For instance, GPER regulates proliferation and migration in breast cancer cells by activating the epidermal growth factor receptor (EGFR) and extracellular signal-regulated kinase 1/2 (ERK1/2) signaling pathways (95, 96). GPER also engages in signal integration with the classical ERα, forming complex regulatory networks that influence cellular fate decisions.

ERα, ERβ, and GPER are not merely in a simple parallel relationship; studies indicate they can form interacting complexes to jointly integrate multiple signaling pathways. The interaction between GPER and classical ER receptors modulates bone and metabolism-related signaling, reflecting the spatiotemporal dependence of estrogen signaling (97). This interaction extends beyond signal transduction to intricate transcriptional crosstalk. For instance, long non-coding RNAs like ERLC1 form positive feedback loops with ERα, enhancing ERα expression and function to influence breast cancer cell proliferation and drug resistance (98).

Estrogen receptors exhibit significant cross-regulation with other nuclear receptors, particularly ERRα. As a class of nuclear receptors, ERRα lacks a natural estrogen ligand but regulates energy metabolism and cell proliferation, forming complementary and competitive relationships with ERα signaling pathways (99, 100). Under certain physiological and pathological conditions, ERRα may regulate or be regulated by ERα, forming complex nuclear receptor signaling networks. Studies have also revealed antagonistic signaling interactions between ERα and retinoic acid receptor α (RARα), bidirectionally regulating complex endocrine disruption effects (101). This cross-regulation among nuclear receptors provides a multi-level regulatory mechanism for modulating cell fate and adapting to environmental changes.

In the context of kidney disease, the complexity of estrogen receptor signaling networks is particularly prominent. ERα, ERβ, and GPER exhibit significant differential expression across distinct renal cell types, and their interplay regulates inflammatory responses, apoptosis, and fibrotic processes in the kidney. The non-genomic pathway mediated by GPER influences glomerular cell proliferation and differentiation by regulating the expression of epidermal growth factor-like factors (EGF-like factors) (102). The interaction between ERα and Yes-associated protein, a key molecule in the Hippo signaling pathway, modulates cell proliferation and fibrosis, thereby affecting the progression of kidney disease (103). Furthermore, the interaction between estrogen receptor signaling and DNA damage repair factors exerts positive effects on renal cell survival and repair mechanisms (90).

### 5.2 Molecular mechanisms of estrogen in renal metabolic regulation

Estrogen plays a complex and multifaceted role in renal metabolic regulation, involving the modulation of energy metabolism and glucose-lipid metabolism, thereby influencing renal physiological function and pathological progression. Estrogen regulates metabolic pathways within renal cells via its receptors, exerting particularly significant effects on FAO and glucose metabolism in tubular cells. Research indicates (104) that PT cells represent the most disease-sensitive cell type in the kidney, with their differentiation state closely linked to metabolic activity. Metabolic pathways such as FAO and oxidative phosphorylation are highly correlated with PT cell differentiation and disease progression. Nuclear receptors like ERRα and PPARα play crucial roles in regulating metabolic gene expression in PT cells, thereby protecting the kidney from disease-induced damage.

In regulating glucose and lipid metabolism, estrogen influences renal lipid homeostasis by modulating the synthesis and degradation pathways of fatty acids and cholesterol. Traditional Chinese medicine *Cuscuta chinensis* exhibits estrogen-like effects, capable of reversing levels of multiple differentially expressed metabolites in ovariectomized rats. These metabolites are involved in lipid metabolic pathways including unsaturated fatty acid biosynthesis, glycerophospholipid metabolism, and arachidonic acid metabolism, demonstrating estrogen’s regulatory capacity over lipid metabolism (105). Decreased FAO activity in kidney disease is often accompanied by lipid accumulation and fibrosis, with key enzymes like carnitine palmitoyltransferase 1α being regulated, affecting mitochondrial β-oxidation of fatty acids. Research indicates that ERβ can interact with PPARα to jointly regulate the expression of FAO-related genes, thereby modulating renal lipid metabolism and mitigating fibrosis progression (25).

On the other hand, metabolic enzymes such as pyruvate kinase M2 (PKM2) also play a crucial role in estrogen-mediated renal pathophysiological processes. As a key glycolytic enzyme, PKM2 not only participates in energy metabolism but also influences the pathological state of renal cells by regulating signaling pathways such as cell proliferation and apoptosis. Although the specific mechanisms underlying PKM2’s role in estrogen-regulated renal function remain incompletely understood, existing research suggests it may participate in regulating metabolic reprogramming of renal cells, thereby affecting disease onset and progression (106, 107).

Additionally, environmental endocrine disruptors such as dioxin-like compounds can enhance binding to and activation of ERα through metabolically activated metabolites, thereby influencing the endocrine environment and metabolic state of the kidney. This suggests that the estrogen-like effects of these metabolites may potentially impact renal metabolic regulation (108). Metabolic reprogramming in the kidney is not only regulated by hormones but also closely linked to changes in cellular mechanical forces. Cytoskeletal proteins like calponin 2 modulate the expression of FAO pathway genes by interacting with ERβ, revealing cross-talk pathways between mechanical signaling and metabolic regulation (25).

### 5.3 Advances in drug development for precision modulation of estrogen signaling

The estrogen signaling pathway exerts complex regulatory effects in various kidney diseases, offering both protective benefits and potential to accelerate pathological progression. The development of SERMs and their novel derivatives, coupled with sex-specific personalized medication strategies, shows great promise in treating kidney diseases.

SERMs are a class of compounds capable of selectively modulating estrogen receptor activity, exhibiting estrogen-like agonist or antagonist effects in different tissues, thereby possessing the ability to “selectively” regulate estrogen signaling. Classic SERMs such as tamoxifen are widely used in breast cancer treatment and have been shown to influence cell proliferation and apoptosis by regulating ER signaling (109). Recently developed oral selective estrogen receptor degraders (SERDs), such as elacestrant and giredestrant, can both inhibit ER signaling and promote ER protein degradation, offering potential to overcome resistance to traditional endocrine therapies (110, 111). As SERMs and SERDs undergo deeper investigation in oncology, their potential applications in renal diseases are gaining attention. Both ERα and ERβ are expressed in renal tissue, and estrogen signaling participates in renal injury and repair processes by regulating apoptosis, inflammatory responses, and oxidative stress pathways. Studies indicate that specific SERMs may exert protective effects by modulating ER signaling in renal tubular epithelial cells, thereby alleviating renal fibrosis and inflammation (52). Natural products and their derivatives, such as phytoestrogenic compounds, also demonstrate potential to influence cell proliferation and apoptosis through selective regulation of ER subtypes, offering novel therapeutic approaches for kidney diseases (112, 113).

It is noteworthy that drug regulation of estrogen signaling exhibits bidirectionality, with certain anti-estrogen drugs potentially causing toxicity or side effects under specific conditions. For instance, anti-estrogen drugs may induce toxic reactions in retinal cells, leading to visual impairment (114, 115). Therefore, developing SERMs and SERDs with high selectivity and low side effects is crucial for achieving precise regulation of estrogen signaling.

Advancements in molecular design techniques, such as structure-based drug design and computer-aided screening, have accelerated the discovery of novel ER ligands and modulators. Screening for potential ER-binding compounds using molecular docking and kinetic simulations provides a theoretical foundation for new drug development (116, 117). The application of proteolysis-targeting chimeras (PROTACs) technology enables novel therapeutics targeting ER degradation, demonstrating significant potential for overcoming resistance (118).

Given the specificity of kidney diseases, future drug development should focus on targeting ER subtypes expressed in the kidney. This approach aims to optimize tissue specificity and signal regulation patterns, enabling precise modulation of estrogen signaling while minimizing systemic side effects. Considering the complexity of inflammatory and fibrotic processes in kidney diseases, exploring multi-target drug design strategies is also warranted.

Sex differences play a significant role in drug metabolism and therapeutic response. As the primary female sex hormone, estrogen exhibits marked variations in levels and signaling activity between males and females, thereby influencing drug pharmacokinetics and pharmacodynamics (119). In women, estrogen levels fluctuate with the menstrual cycle, pregnancy, and menopausal status, introducing dynamic variations within individuals and further complicating treatment strategies.

In the treatment of kidney disease, tailoring medication regimens to account for sex differences can enhance therapeutic efficacy and reduce the risk of adverse reactions. Certain drugs targeting ER may demonstrate more pronounced efficacy in female patients, while male patients may require dose adjustments or alternative treatment options (52). The expression and activity of drug-metabolizing enzymes, such as the cytochrome P450 (CYP) family, are also regulated by sex and estrogen levels, influencing drug absorption, distribution, metabolism, and excretion (119).

Based on this, clinical trial designs should fully incorporate sex as a variable to evaluate differences in patient response to estrogen-modulating drugs between male and female patients. This approach enables optimized dosing and administration regimens, continuous monitoring of estrogen levels in patients, and integration with genomic and metabolomic data—ultimately achieving more precise medication management.

In addition to sex, factors such as age, endocrine status, and concomitant medications must also be comprehensively considered. Postmenopausal women experience decreased estrogen levels, potentially requiring different hormone replacement therapy regimens or adjusted doses of modulators (120). Research on individualized dosing for pediatric, pregnant, and elderly patients remains insufficient and urgently needs to be strengthened. In the future, integrating artificial intelligence and big data technologies to establish predictive models for drug responses based on sex and endocrine status will facilitate precision medicine for estrogen signaling-modulating drugs, thereby enhancing overall treatment outcomes for patients with kidney disease (121).

Thus, in the treatment of kidney diseases, SERMs and their novel derivatives demonstrate significant promise, with sex-specific personalized medication strategies being key to achieving precision therapy. Future drug development should focus on enhancing targeting and safety while integrating sex and endocrine dynamics to advance clinical translation.

### 5.4 Prospects of gene editing and molecular targeting technologies in estrogen regulation

With the rapid advancement of gene editing technologies, particularly the widespread application of the clustered regularly interspaced short palindromic repeats/CRISPR-associated protein 9 (CRISPR/Cas9) system, powerful tools have emerged for precisely regulating estrogen and its receptors in studying the mechanisms underlying kidney diseases. CRISPR/Cas9 enables both single-gene knockout or mutation and multi-gene co-editing, significantly enriching our understanding of estrogen synthesis pathways and signaling networks.

In studies of sex determination and differentiation in fish, multiple knockouts of estrogen synthesis-related genes (e.g., cyp19a1a, cyp19a1b) and estrogen receptors (esr1, esr2a, esr2b) validated the critical role of estrogen receptor subtypes in female sex determination (122). Conditional gene editing techniques combining CRISPR/Cas9 with HoxB8 pluripotent progenitor cells enable efficient, precise gene regulation in immune cells, providing a model for studying estrogen’s role in immune regulation and inflammatory responses in kidney diseases (123).

In tumor research, gene editing has revealed that acquired mutations in the estrogen receptor 1 (ESR1) gene constitute a key mechanism for resistance to endocrine therapy. Point mutations and fusion genes enhance the receptor’s transcriptional activity, while knocking down the critical transcription factor X-box binding protein 1 reverses this drug resistance, offering new insights for targeted therapy (124). Similarly, CRISPR/Cas9-mediated gene knockout techniques have been employed to investigate the functions of key regulatory factors in breast cancer, demonstrating that precise genetic regulation can effectively influence estrogen signaling pathways and their downstream effects (125).

Additionally, PROTAC technology, as a chemical alternative strategy to gene editing, can rapidly and reversibly reduce ERα protein levels while avoiding embryonic lethality or compensatory mechanisms associated with gene knockout. It has been demonstrated to effectively modulate estrogen function in animal models, suggesting its potential for clinical applications (126).

In the future, within the framework of precision medicine, combining gene editing and molecular targeting technologies will enable precise regulation of estrogen signaling pathways. By identifying mutations or epigenetic states of estrogen receptors and their core regulatory factors in patients with kidney disease, tailored gene editing strategies or small-molecule modulators can be designed to achieve personalized treatment. Concurrently, multi-omics integration analysis and AI-assisted gene network prediction will further advance the application of gene editing technologies in estrogen regulation. This will optimize therapeutic outcomes, reduce side effects, and promote precision diagnosis, treatment, and improved prognosis for kidney disease patients (127).

## 6 Clinical evidence and sex differences in the impact of estrogen on kidney diseases

### 6.1 Correlation analysis of estrogen levels and renal function in large-scale clinical cohorts

In recent years, with the rising incidence of kidney disease, investigating the correlation between sex hormones—particularly estrogen—and renal function has become a significant research focus. Large-scale clinical cohort studies provide extensive data support for elucidating estrogen’s impact on renal function across diverse populations, revealing its complex physiological and pathological effects.

Statistical analysis of estrogen and renal function parameters across different sexes and age groups indicates that women, particularly those of reproductive age, exhibit more stable renal function compared to men. This is closely associated with the protective effects of estrogen (128). A systematic review indicated that postmenopausal women, due to declining estrogen levels, are prone to kidney diseases such as kidney stones. By analyzing eight studies, this research confirmed a positive correlation between serum estrogen levels and renal function. Furthermore, estrogen reduces the risk of kidney stone formation by downregulating the activity of the kidney-specific anion transporter SLC26A6, thereby decreasing oxalate transport (129). Another retrospective cohort study in transgender individuals found that when renal function was estimated based on birth sex, no significant changes were observed in the estrogen-treated group. However, when estimated based on sex identity, renal function indicators showed a declining trend in the estrogen group, suggesting estrogen’s impact on renal function and highlighting the critical importance of estimation method selection for interpretation (130). Prospective multicenter observational studies further demonstrated that estrogen exerts renal protective effects by improving renal hemodynamics and filtration function, whereas testosterone may adversely affect renal function (34).

From the perspective of physiological hormone level changes, it is common for postmenopausal women to experience reduced kidney function due to decreased estrogen levels. A cross-sectional study involving 2,540 postmenopausal women revealed elevated follicle-stimulating hormone (FSH) levels were associated with increased serum creatinine and decreased eGFR. This suggests that high FSH levels may be an independent risk factor for renal impairment, with this association intensifying with advancing age (12). In CKD patients, reduced estrogen receptor expression correlates with disease progression, particularly in IgAN patients where decreased renal ERα expression is closely associated with worsening renal function and poor prognosis (32).

### 6.2 Current status and challenges in sex-specific diagnosis and treatment of kidney diseases

Significant sex differences exist in the epidemiology of CKD. Global data show females have a higher overall CKD prevalence than males, yet males progress faster to ESRD with poorer outcomes (131, 132). Carrero et al. highlights that this higher female prevalence is not simply due to greater disease susceptibility, but results from a combination of longer life expectancy, sex-specific risk factors, and biases inherent in renal function estimation equations (133).

The age-related cumulative effect is a key driver. Women live longer than men, and as CKD is an age-related disease, the larger elderly female population elevates the detected prevalence. Additionally, females face sex-specific risk factors, including pregnancy-related injury, recurrent urinary tract infections, autoimmune nephropathies, and metabolic disorders (14, 134, 135). Importantly, the commonly used CKD-EPI and MDRD equations, developed primarily from Western cohorts, tend to underestimate eGFR in women due to lower muscle mass and baseline creatinine, leading to possible overdiagnosis (133). Conversely, this bias may also cause early-stage disease to be missed in younger women. The interplay of these factors, compounded by the loss of estrogen’s renoprotective effects after menopause, contributes to the observed epidemiological pattern of higher female prevalence but slower disease progression (14).

In male kidney disease, the loss of estrogen’s protective effects manifests as metabolic disorders, heightened inflammation, and vascular dysfunction, contributing to faster functional decline, abnormal uric acid metabolism, and high cardiovascular risk (14, 136–138). For instance, male proximal tubular epithelial cells show higher mitochondrial respiration and oxidative stress, predisposing them to apoptosis (136). Hyperuricemia in males is significantly linked to adverse renal outcomes, a relationship not observed in females (138). Therapeutically, some studies suggest that anti-androgen therapy or estrogen replacement may improve renal function and hemodynamic parameters in male patients, highlighting the potential value of estrogen-related pathways (34, 137). However, response to such hormone therapy is complex and may lead to metabolic side effects, warranting individualized assessment.

Clinically, sex-specific considerations are crucial across the entire care spectrum. Women with kidney disease experience a higher burden of cardiovascular complications (139, 140), more psychological distress (141), and lower disease awareness (142), which hinders early intervention. Treatment needs must account for hormonal fluctuations across the menstrual cycle, pregnancy, and menopause (143). For men, although endogenous estrogen levels are low, the expression of estrogen receptors in the kidney influences renal health (14, 137). Addressing these sex-specific needs—through enhanced disease education, optimized hormonal management, psychosocial support, and equitable resource allocation—is essential for achieving precision medicine and improving clinical outcomes in kidney disease.

### 6.3 Potential of estrogen and its receptors as biomarkers in kidney diseases

The altered expression of ERs in kidney disease demonstrates their potential as biomarkers. Studies have shown that ER expression levels exhibit significant differences across distinct stages and pathological states of kidney disease, closely correlating with disease progression and prognosis. For instance, in kidney lesions associated with DICER1 tumor susceptibility syndrome, ER expression is prominent in the stromal cells of pediatric cystic nephroma and early tumor types. However, as lesions progress toward more malignant and parenchymal forms, ER expression gradually diminishes, accompanied by increased expression of the preferentially expressed antigen in melanoma. This suggests a negative correlation between ER expression levels and renal pathological status and malignancy grade (144), indicating potential value for disease staging and prognostic assessment.

Research on renal cancers such as papillary renal cell carcinoma reveals that hormone receptor-related pathways, including estrogen receptor signaling, participate in tumor cell cycle regulation, DNA damage repair, and the activation of multiple signaling pathways. The spindle and kinetochore-associated protein 3 (SKAP3) gene is closely associated with these pathways. Elevated SKAP3 expression correlates with reduced overall survival in patients, suggesting that estrogen receptor activity is closely linked to renal cancer progression and prognosis (145). These findings support the potential of estrogen receptor expression levels as a biomarker for renal tumors.

In the development of sex-specific biomarkers, researchers have increasingly integrated sex as a critical variable due to the sex-specific expression of ERs and their differential roles in sex-specific kidney diseases. For instance, the mechanisms underlying ER activation or inhibition in female kidney diseases differ from those in males. The GPER agonist G1 demonstrated protective effects in a male mouse CKD model, improving renal function metrics and reducing fibrosis and inflammation, suggesting that ER-targeted therapies may require sex-specific adjustments (146). In autoimmune kidney diseases like SLE, estrogen-regulated miRNAs and their signaling via exosomes are considered key markers of disease activity and progression. These signaling pathways also exhibit marked sex differences, further underscoring the importance of integrating sex factors into biomarker research (81).

## 7 Future research directions and challenges in estrogen regulation of kidney disease

### 7.1 Application of multi-omics technologies in investigating estrogen’s renal action mechanisms

With the rapid advancement of omics technologies, multi-omics approaches have become essential tools for unraveling the complex mechanisms of estrogen’s role in kidney diseases. By integrating transcriptomic, proteomic, and metabolomic data, researchers can systematically map estrogen-regulated molecular networks, further elucidating functional differences and action pathways across distinct renal cell types.

Transcriptomics technologies, particularly RNA sequencing (RNA-seq), have been extensively applied to elucidate the expression regulation of estrogen-related genes in the kidney. Studies indicate that in mouse proximal tubule cells, sex-specific gene expression differences are regulated by gonadal hormones, with the androgen receptor (AR) directly mediating gene activity regulation while the estrogen receptor also participates in indirect regulatory processes (147). By integrating RNA-seq with assay for transposase-accessible chromatin with high-throughput sequencing (ATAC-seq) and proteomic mass spectrometry, the study identified over 7,000 differentially accessible DNA regions in proximal tubule cells. These regions were closely associated with sex-biased gene expression, and their regulatory mechanisms involved the coordinated action of multiple transcription factors rather than simple direct binding by hormone receptors (148). Spatiotemporal transcriptomics and multi-omics analyses revealed distinct patterns of sex-biased gene expression across different regions of the renal cortex and outer medulla, with these differences exhibiting dynamic changes over time (149). These variations in gene expression and regulation provide a molecular basis for understanding estrogen’s role in renal sex differences.

The application of proteomics enables researchers to identify protein expression changes associated with estrogen signaling and their functional significance in kidney disease. Whole-kidney proteomics analysis revealed that microfibril-associated protein 2, a key core matrix component during renal repair, is lost. This loss can lead to ERβ-mediated transcriptional suppression, thereby affecting the expression of key metabolic enzymes and ultimately exacerbating AKI (150). In a hyperuricemic nephropathy model, combined proteomics and transcriptomics revealed that the SGLT2 inhibitor dapagliflozin promotes uric acid excretion and alleviates renal interstitial fibrosis by activating the ERRα-organic anion transporter 1 axis (151). These findings underscore the critical role of proteomics in elucidating estrogen signaling pathways and their downstream effector molecules.

The introduction of metabolomics has further enriched our understanding of estrogen’s role in regulating renal metabolism. Metabolomics studies have revealed that metabolic pathways such as fatty acid metabolism, amino acid metabolism, and steroid hormone biosynthesis are significantly disrupted during kidney diseases, particularly renal fibrosis (19, 50). Kidney-specific AMPK activity is closely correlated with estrogen levels, regulating metabolic reprogramming within tubular cells and conferring enhanced nutritional stress resistance in females (50). Plant extracts from traditional Chinese medicine exhibit estrogen-like renal protective effects by modulating lipid and amino acid metabolism, with metabolomic analyses elucidating their underlying mechanisms (105, 152).

In terms of single-cell sequencing technology, single-cell RNA sequencing provides fine cellular resolution for analyzing renal cell heterogeneity and estrogen effects. By analyzing the transcriptional expression profiles of distinct renal cell populations in healthy and diseased states, researchers identified proximal tubule cells as the renal cell type most susceptible to sex hormone influences, with significant alterations in their differentiation and metabolic characteristics under pathological conditions (104, 153). In single-cell transcriptomes from AKI patients’ kidneys, altered expression of estrogen signaling pathway genes was closely associated with cell apoptosis and inflammatory responses, suggesting estrogen’s potential regulatory role in renal injury repair (153). Furthermore, single-nucleus RNA sequencing + single-nucleus ATAC sequencing (snRNA-seq + snATAC-seq) has demonstrated powerful capabilities in deciphering hormone mechanisms. Using this approach, researchers achieved precise localization at both the cellular (proximal tubule) and chromatin levels, ultimately revealing that the establishment of renal sexual dimorphism primarily relies on direct transcriptional regulation by the androgen receptor (AR), with a relatively limited contribution from ERα (147).

Future studies combining spatial omics with multimodal data integration analysis will be instrumental in further unraveling the precise regulatory mechanisms of estrogen across different kidney cell types, thereby advancing the development of personalized therapies.

### 7.2 Systematic incorporation of sex differences in animal models and clinical research

In both basic and clinical research on kidney diseases, the systematic incorporation of sex differences is crucial for elucidating disease pathogenesis and optimizing personalized treatment strategies. Traditional studies have often prioritized male animal models and male patients, overlooking the unique role of females in pathophysiological processes—particularly estrogen-regulated renal protective mechanisms. To advance research designs incorporating female animal models and female clinical patients, current studies emphasize the need to include sex as an independent variable in experimental design and data analysis. This approach enables systematic evaluation of how sex influences disease progression and treatment response.

Promoting research designs using female animal models can more comprehensively simulate the dynamic changes in female physiological states and hormone levels. Studies indicate that GPER exerts protective effects against cardiovascular and renal diseases across various experimental animal models. Its mechanisms include interactions with aldosterone and endothelin-1 signaling pathways, promotion of nitric oxide release, and mitigation of oxidative stress, inflammation, and immune cell infiltration (154). These effects are particularly pronounced in female models, suggesting the potential value of estrogen and its receptors in slowing renal disease progression. Research using female animals should also account for factors such as the physiological cycle and menopausal status to thoroughly investigate the specific impacts of hormonal changes on renal function and disease phenotypes.

In clinical research, systematically including female patients is equally critical. Existing data indicate sex differences in the incidence and progression of CKD, with women typically exhibiting higher CKD prevalence than men, while men experience more pronounced GFR decline and cardiovascular mortality (76, 155). Furthermore, female patients experience higher rates of cardiovascular events during dialysis treatment, while males are more prone to early complications such as anemia and secondary hyperparathyroidism (76). These findings underscore the necessity of considering sex as a standard variable in clinical trial design and treatment protocol development, thereby advancing precision medicine.

The standardized application of sex as a variable in kidney disease research is equally indispensable. Sex differences are reflected in epidemiological characteristics of diseases as well as in molecular mechanisms, drug metabolism, and therapeutic efficacy disparities. Estrogen protects women from hypertension and associated renal injury by regulating ENaC and aquaporin 2 expression to maintain extracellular fluid volume homeostasis (14, 39). Androgen receptor (AR)-mediated gene expression regulation is prominent in male kidneys, and environmental factors such as dietary restriction can exert sex-specific effects on it (147). Therefore, preclinical and clinical studies should adopt standardized approaches for handling the sex variable to ensure data comparability and scientific rigor.

Sex differences are equally pronounced in drug research. Taking the SERM tamoxifen as an example, studies indicate it effectively alleviates renal fibrosis in both males and females, yet individual responses exhibit sex-specific variations (51). Novel antidiabetic drugs such as SGLT-2 inhibitors and glucagon-like peptide-1 (GLP-1) receptor agonists exhibit sex-related efficacy differences in DKD treatment, suggesting that future clinical trials should incorporate sex-stratified analyses (156). These advances compel researchers and clinicians to systematically incorporate sex factors when designing studies and formulating treatment strategies, advancing precision medicine in kidney disease.

### 7.3 Comprehensive analysis of estrogen regulation and multifactorial interactions in kidney disease

The role of estrogen in kidney disease is complex and multidimensional. Beyond directly influencing renal function through receptor-mediated signaling pathways, it also intertwines with environmental, genetic, and metabolic factors, forming a multifactorial interaction network. Environmental factors such as inflammation, oxidative stress, and metabolic disorders significantly impact the activity and effects of estrogen signaling pathways. For instance, in DKD, ERRα exerts protective effects by maintaining mitochondrial integrity in proximal tubule cells. Its expression is regulated by the E3 ubiquitin ligase RBBP6; RBBP6-mediated degradation of ERRα exacerbates mitochondrial damage and accelerates disease progression, suggesting cross-talk between metabolic abnormalities and estrogen signaling imbalance (66). Localized renal inflammation and immune cell infiltration have been reported to interact directly or indirectly with the ERα/ERβ signaling pathways in multiple kidney diseases. For instance, the coupling of estrogen receptors with the TLR4 signaling pathway modulates the expression of the inflammatory cytokine IL-1β, influencing the inflammatory process in sepsis-induced AKI (40). Regarding genetic factors, ERα gene polymorphisms have been implicated in susceptibility and prognosis across multiple renal disorders, influencing kidney function through multiple signaling pathways including regulation of tubular ion channels, inflammatory responses, and apoptosis (8). The interaction between sex chromosomes and sex hormones also provides a genetic basis for sex differences in kidney disease (14). Among metabolic factors, the sex-specific regulation of renal AMPK activity is particularly critical. The estrogen-regulated AMPK activity in females exhibits a protective effect against DKD, and differential expression of metabolic pathways leads to varying susceptibility and progression rates of kidney disease between sexes (50). The complex interplay of environmental, genetic, and metabolic factors with estrogen signaling is integral to both the onset and progression of kidney disease and the modulation of treatment response and prognosis, highlighting the need for multifactorial integrated analysis in precision medicine.

With the support of big data and artificial intelligence (AI) technologies, constructing comprehensive risk assessment models has become feasible. By integrating genomic, transcriptomic, metabolomic, and clinical phenotypic data, machine learning algorithms can identify key variables and interaction patterns influencing kidney disease risk and progression. For instance, weighted gene co-expression network analysis has identified critical gene modules and transcription factors in chronic transplant nephropathy, offering new insights into disease mechanisms (157). Combining molecular markers of estrogen-related signaling pathways—such as GPER expression levels, ERα polymorphisms, AMPK activity, and inflammatory factor expression—enables the construction of multidimensional risk prediction models to support personalized clinical treatment decisions (50, 154). AI-driven drug target screening and molecular docking technologies have been applied to studies of traditional Chinese medicines like *Cornus officinalis* for treating DKD, revealing interactions between estrogen signaling pathways and multiple metabolic and inflammatory pathways (158). In gender-diverse patient populations, such as transgender individuals, AI models based on the effects of hormone therapy on renal function can also assist in optimizing renal function estimation, enhancing the accuracy of clinical management (34, 130). In the future, as data volume and diversity continue to expand, integrating multifactorial information—including environmental exposures, genetic background, endocrine status, and lifestyle—to construct dynamic kidney disease risk assessment systems through deep learning and network analysis will significantly advance the precision prevention and personalized treatment of estrogen-related kidney diseases. This will close the loop from mechanism exploration to clinical translation.

## 8 Conclusion

Estrogen exerts a complex and paradoxical double-edged sword effect in kidney diseases, a characteristic that poses significant challenges for its research and clinical application. As a sex hormone with multiple regulatory functions, estrogen influences both physiological and pathological processes in the kidney through various receptors and signaling pathways. It can exert protective effects, such as anti-inflammation, anti-fibrosis, and hemodynamic regulation, yet may also promote disease progression under certain pathological conditions. This complexity underscores that estrogen cannot be simplistically categorized as either a “beneficial” or “harmful” factor. Instead, we must delve into the subtle differences and condition-dependent mechanisms underlying its actions.

Recent studies have significantly highlighted the crucial role of sex differences in the onset and progression of kidney disease. Male and female patients exhibit marked disparities in disease presentation, progression rates, and treatment responses, partly attributable to differences in estrogen and its receptor-mediated signaling pathways.

In practical application, balancing the protective effects of estrogen with its potential pro-disease risks has become a critical issue in clinical treatment design. For female patients with certain kidney diseases, moderate regulation of estrogen levels may yield significant therapeutic benefits. Conversely, for male patients or those in specific pathological states, careful assessment of the risks and benefits associated with estrogen-related therapies is essential.

To maximize the protective effects of estrogen, future research and clinical practice should focus on precisely regulating estrogen signaling. At the micro level, this involves utilizing single-cell multi-omics technologies to decipher the dynamic expression profiles of ER subtypes in specific renal cell types, and developing GPER-targeted agonists—which may represent one of the most promising paths to achieve renal protection without inducing systemic side effects. At the macro level, systematic analysis from a sex perspective must be strengthened, particularly across different age groups, hormonal statuses, and kidney disease types. By deeply integrating precision medicine principles with multi-omics data and advancing the development and clinical application of novel modulators, new avenues for achieving sex-specific precision therapy can be forged. Ultimately, through precise regulation of estrogen signaling and sex-specific therapeutic strategies, we aim to enhance the comprehensive management of kidney disease patients and advance medical progress as a whole.
